# Retrocardiac lung hernia after thoracic esophagectomy: report of a rare case

**DOI:** 10.1186/s40792-015-0058-9

**Published:** 2015-07-14

**Authors:** Akinobu Furutani, Masahiro Niihara, Keisuke Kawamorita, Shoji Takahashi, Yasuhisa Ohde, Yasuhiro Tsubosa

**Affiliations:** Division of Esophageal Surgery, Shizuoka Cancer Center Hospital, 1007 Shimonagakubo, Nagaizumi-cho, Sunto-gun, Shizuoka 411-8777 Japan; Division of Thoracic Surgery, Shizuoka Cancer Center Hospital, 1007 Shimonagakubo, Nagaizumi-cho, Sunto-gun, Shizuoka 411-8777 Japan

**Keywords:** Esophageal surgery, Lung hernia, Mediastinum

## Abstract

A retrocardiac lung hernia is an extremely rare complication after esophagectomy. A 56-year-old man was admitted to our hospital with advanced middle thoracic esophageal cancer and a giant bulla at the apical portion of the right lung. Since it appeared that dissection of the upper mediastinum would most likely require resection of the right bulla, a two-stage operation for esophageal cancer was planned. During the first-stage operation, thoracic esophagectomy and resection of the right giant bulla were performed. Fourteen days after the first-stage operation, the patient underwent laparotomy as the second-stage operation to reconstruct a narrow gastric tube via a retrosternal route. After the second-stage operation, the inflammatory reaction was prolonged. Therefore, a thoracoabdominal computed tomography scan was performed, showing retrocardiac pulmonary atelectasis. The patient was diagnosed with a retrocardiac left lung hernia in which the left lower lobe was displaced into the right thoracic cavity. Because the inflammatory reaction was due to effects of the lung hernia, a repair operation was performed via a left seventh intercostal thoracotomy. At thoracotomy, the left basal segment of the lung was atelectatic and reddish and had herniated into the right thoracic cavity through an opening between the aorta and pericardium. The herniated lung tip adhered strongly to the subcarina, and synechiotomy was performed. We believe that simultaneous removal of the right giant bulla with esophagectomy was the important cause of this complication.

## Background

Retrocardiac lung hernia is an extremely rare complication of esophagectomy that requires surgical repair, and to date, there has been only one published report describing such a complication [1]. We encountered a case of retrocardiac lung hernia, and because it was an extremely rare case, we here report the details.

## Case presentation

**Fig. 1:**
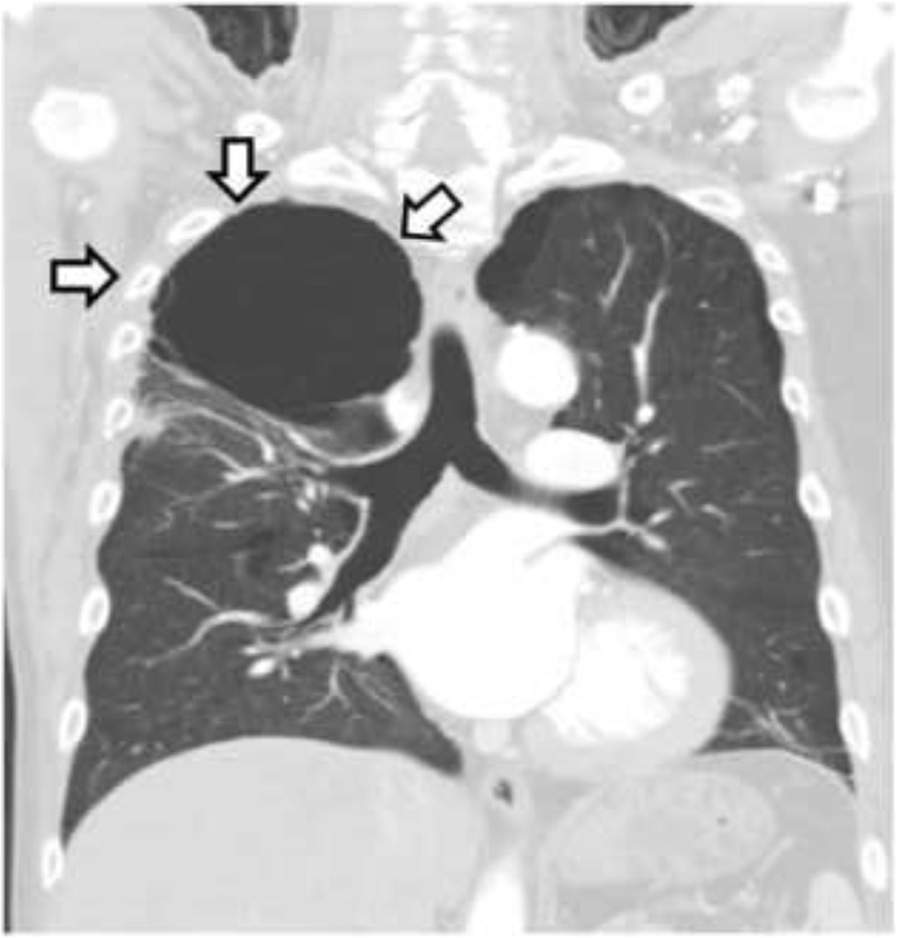
A giant 9-cm lung bulla is observed in the apical portion of the right lung

A 56-year-old male patient was referred to our hospital after being diagnosed with thoracic esophageal cancer. Upper gastrointestinal endoscopy revealed a 9-cm, type 2 tumor in the middle thoracic esophagus. Histological examination of biopsy specimens confirmed squamous cell carcinoma. Computed tomography (CT) of the chest revealed a thick wall around the middle thoracic esophagus, a mediastinal lymph node metastasis, and a giant 9-cm bulla at the apical portion of the right lung (Fig. 1). The clinical stage was IIIa (T3, N1, M0) according to the Union for International Cancer Control, seventh edition. Since it appeared that dissection of the upper mediastinum would most likely require resection of the right giant bulla, a two-stage operation for esophageal cancer was planned, as it is our hospital’s policy to plan a two-stage operation for risky cases.

During the first-stage operation, right thoracic subtotal esophagectomy and cervicothoracic lymph node dissection were performed under general and epidural anesthesia. There was marked adhesion between the giant bulla and the chest wall, and bullectomy was performed. During dissection of the lower thoracic esophagus, an approximately 3-cm pleural window was made in the left mediastinal pleura, which led to a connection between the right and left thoracic cavities. The thoracic esophagus was dissected above the diaphragm, the mediastinal pleural window was not closed, a drainage tube was inserted from the right side of the thorax to the apical portion of the right lung, and the chest was closed. Esophagostomy was performed at the neck, while jejunostomy was performed by laparotomy. After the surgery, aspiration via a drainage tube at −10 cmH_2_O was performed continuously, and the drainage tube was removed on postoperative day 5. The patient’s course after the first-stage operation was uneventful, and no clear respiratory symptoms occurred. Although we gave the patient loxoprofen 600 mg/day and buprenorphine 0.4 mg/day as analgesic drugs in addition to epidural anesthesia, the patient complained of postoperative pain and was unable to expectorate sputum sufficiently.

**Fig. 2:**
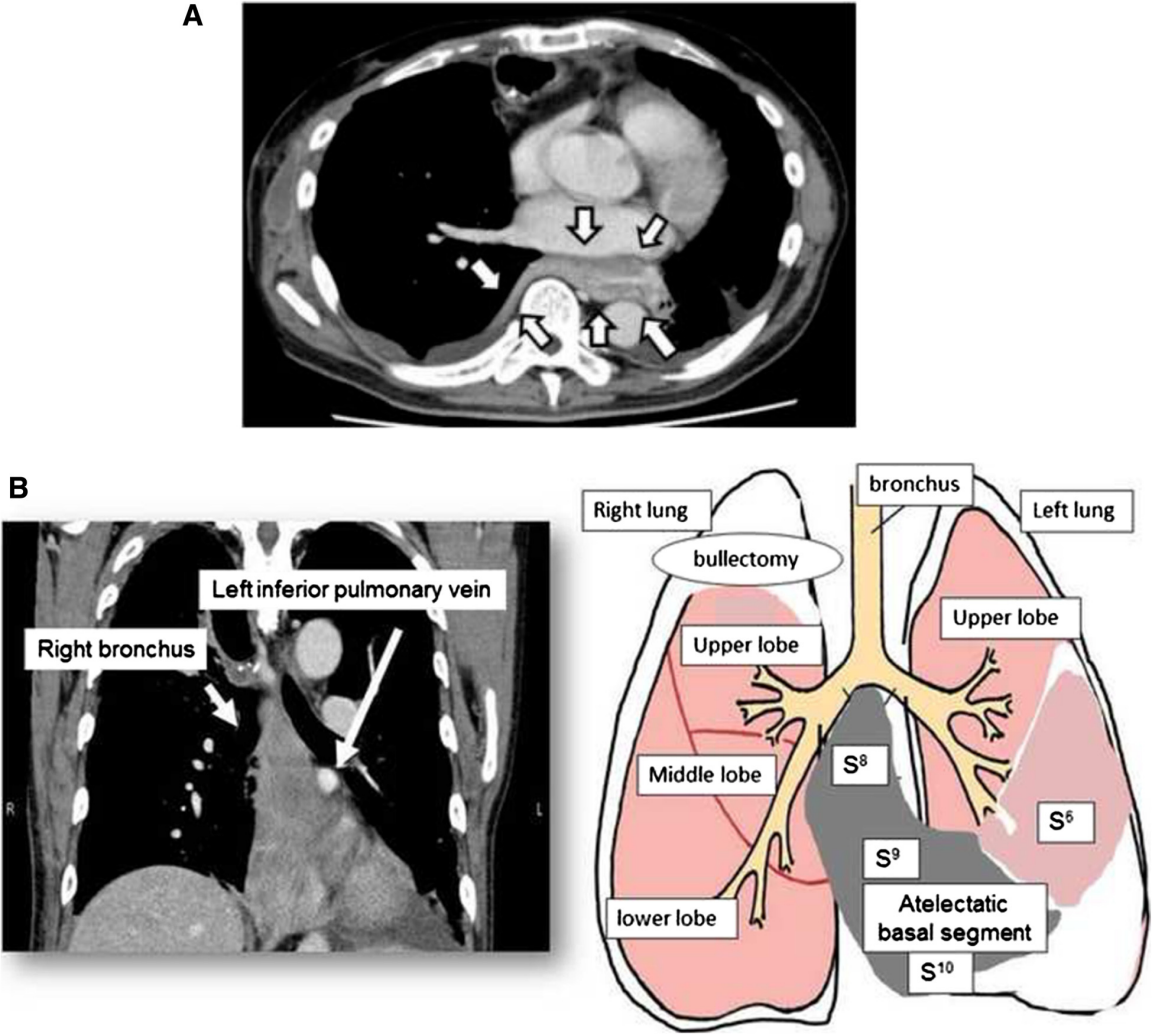
**a** Atelectatic lung is observed between the heart and aorta. Blood flow from the left pulmonary artery to the atelectatic lung is observed. **b** The atelectatic basal segment is displaced into the subcarina

**Fig. 3:**
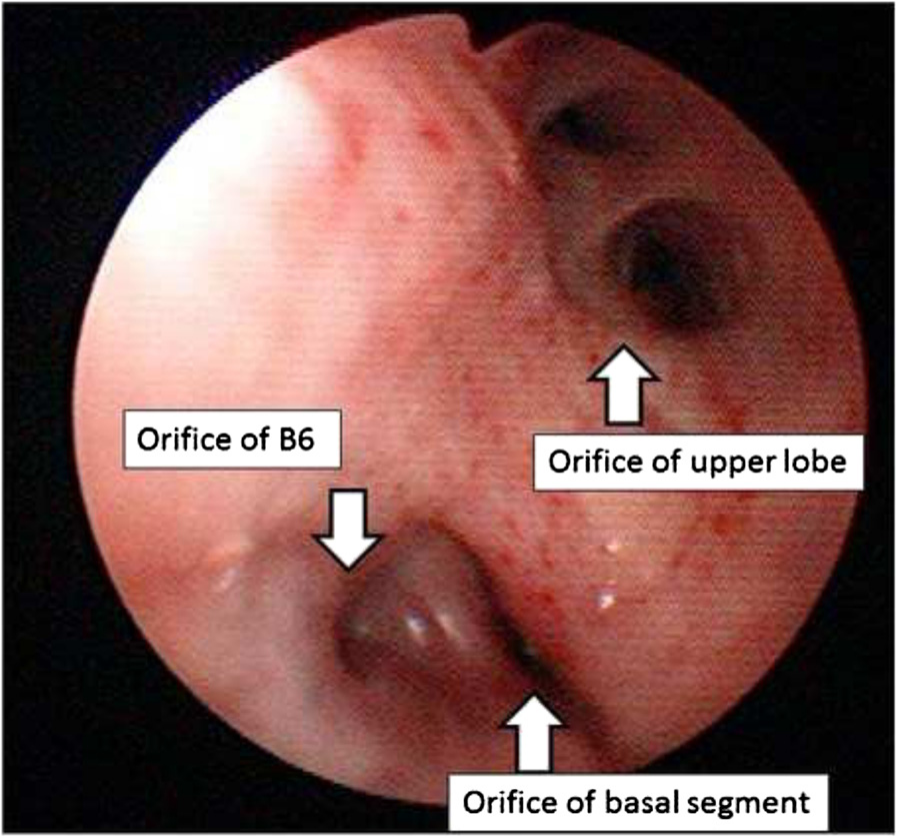
Bronchial occlusion was observed at the base of the left lung, and although it was possible to pass the bronchoscope, the occlusion was not resolved

Fourteen days after the first-stage operation, the patient underwent laparotomy as the second-stage operation to reconstruct a narrow gastric tube via a retrosternal route. The abdominal esophagus was dissected from the esophageal hiatus. The esophageal hiatus was sutured. Neither dyspnea nor hypoxemia was observed after the second-stage operation. PaO_2_ of the patient was 71 mmHg in room air. However, an inflammatory reaction associated with a white blood cell count of about 11,000/μl and C-reactive protein levels of 6.21 mg/dl continued; on day 27 after the first operation, thoracoabdominal CT was performed, showing retrocardiac pulmonary atelectasis. Blood flow from the left pulmonary artery to the atelectatic lung was observed, and the patient was diagnosed with a retrocardiac left lung hernia in which the left lower lobe was displaced into the right thoracic cavity (Fig. 2a, b). At bronchoscopy, bronchial occlusion was observed at the base of the left lung, and although it was possible to pass the bronchoscope, the occlusion was not resolved (Fig. 3). This was thought to be due to bending of the bronchus. Assuming that the inflammatory reaction was due to the effect of the lung hernia, a diagnostic thoracoscopy of the left chest cavity was performed.

**Fig. 4:**
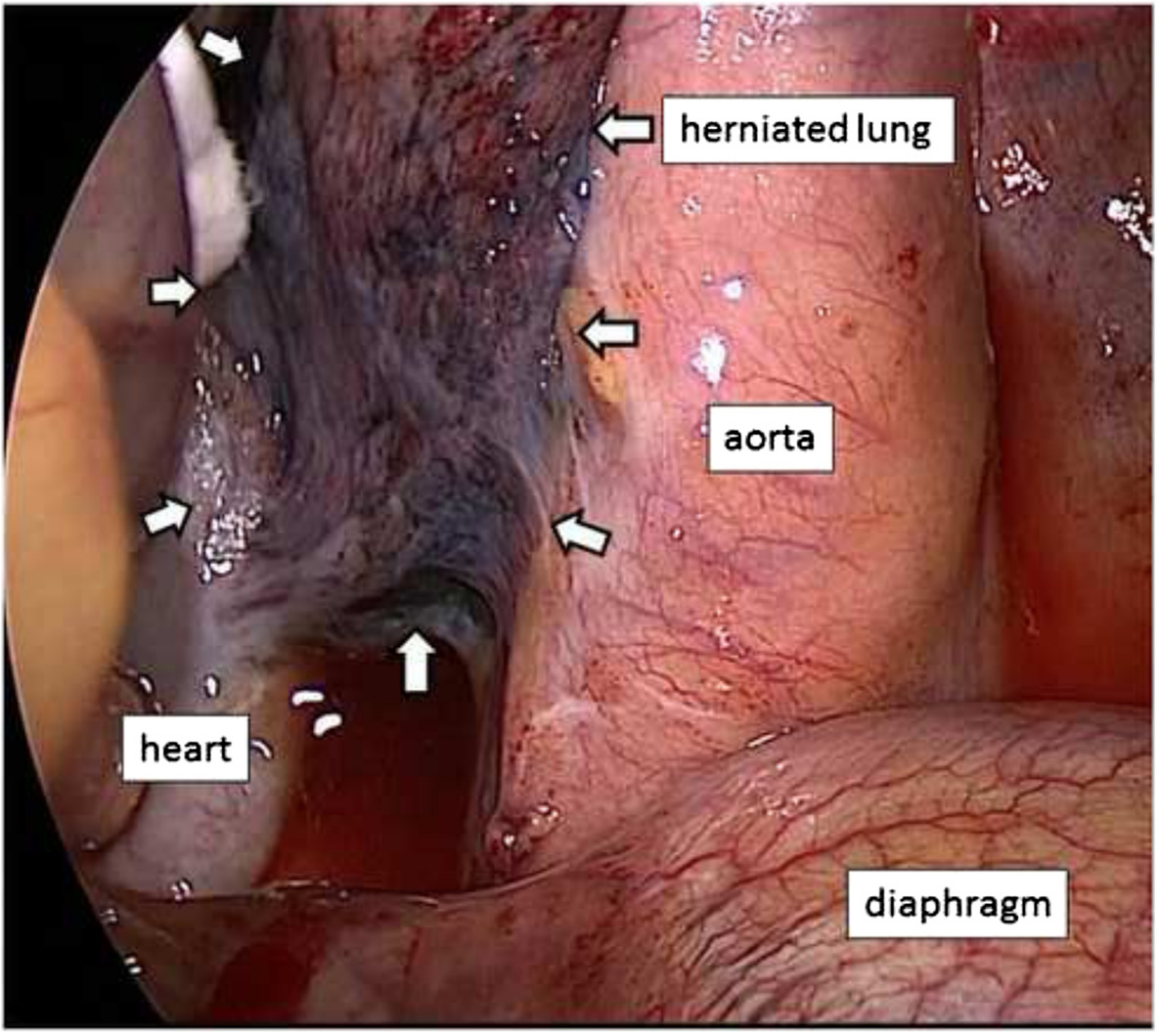
The left basal lung segment was atelectatic and reddish and had herniated into the right thoracic cavity from an opening between the aorta and pericardium

At diagnostic thoracoscopy, the left basal segment of the lung was atelectatic and reddish and had herniated into the right thoracic cavity through an opening between the aorta and pericardium (Fig. 4). The herniated lung tip adhered strongly to the subcarina, and a repair operation by thoracotomy was necessary. At a left seventh intercostal thoracotomy, synechiotomy was performed. The left basal segment was re-integrated into the left thoracic cavity, and neither ischemia nor necrosis was observed. The hernial orifice was closed by suturing the mediastinal pleura. A drainage tube was inserted into the left apical portion of the pleural cavity, while another was inserted into the portion of the mediastinum into which the lung had herniated, both via the left thoracic cavity, and the chest was then closed. The patient’s postoperative course was uneventful. The patient is currently being followed as an outpatient, and no recurrence of the lung hernia has been observed.

### Discussion

In the present case, the mechanism of retrocardiac lung hernia development was likely due primarily to the resection of the giant bulla in the apical portion of the right lung. It is possible that, because the volume of the right thoracic cavity was reduced by the removal of the giant bulla, the left lung intruded into the right thoracic cavity to compensate for the reduced volume. In addition, the complication may have occurred because the contralateral pleura was opened at the time of dissection of the thoracic esophagus, as often happens, and because the left lung developed atelectasis as a consequence of insufficient expectoration of sputum due to postoperative pain. Thus, the left lung was likely displaced into the right thoracic cavity as a result of the combined effects of all of the above possible causes, resulting in the retrocardiac lung hernia.

Regarding continuous drainage in the thoracic cavity, we believe that the lung displacement occurred toward the side of continuous drainage. As we did in the present case, continuous drainage in the right thoracic cavity was performed by John et al. [1] in the published case of retrocardiac lung hernia, and in both cases, the left lung herniated into the right thoracic cavity. The pressure in the thoracic cavity on the side of the drainage became slightly lower than that in the contralateral thoracic cavity, and this may have caused the herniation to occur more easily.

In the present case, gastric tube reconstruction was performed via the retrosternal route. Therefore, the space created at the site of the esophagus could also have contributed to this complication. On the other hand, in the case of John et al. [1], the lung hernia developed between the heart and a gastric tube that was reconstructed via a retromediastinal route. Therefore, it remains unclear whether the reconstruction route could be the cause of a retrocardiac lung hernia.

CT and bronchoscopy are useful for the diagnosis of retrocardiac lung hernia. CT, in particular, should demonstrate an atelectatic lung in the retrocardiac position, making it easy to diagnose the hernia. During bronchoscopy, an occlusion due to the bending of the bronchus of the herniated lung is expected. Characteristically, although the bronchoscope can pass through the occluded part, it will not resolve the occlusion.

In our case as well as in that of John et al. [1], the patient needed surgical repair. In our case, because of the adhesion of the herniated lung to the sites of esophagectomy and cervicothoracic lymph node dissection, spontaneous recovery was unlikely. Thus, the most effective treatment for retrocardiac lung hernia may be synechiotomy via thoracotomy.

## Conclusions

An extremely rare case of a retrocardiac lung hernia that developed after esophagectomy was described, which may have been caused by the removal of a giant bulla and the creation of a mediastinal pleural window. To prevent the development of a retrocardiac lung hernia, it is important to securely close the pleura after opening. Moreover, when the patient is unable to expectorate sputum sufficiently due to postoperative pain, adequate analgesia may be necessary to prevent lung atelectasis.

## Consent

Written informed consent was obtained from the patient for the publication of this case report and any accompanying images. A copy of the written consent is available for review by the Editor-in-Chief of this journal.
